# Epigenetic Regulation in Pathology of Atherosclerosis: A Novel Perspective

**DOI:** 10.3389/fgene.2021.810689

**Published:** 2021-12-15

**Authors:** Haishuang Tang, Zhangwei Zeng, Chenghao Shang, Qiang Li, Jianmin Liu

**Affiliations:** Department of Neurosurgery, Changhai Hospital, Naval Military Medical University, Shanghai, China

**Keywords:** atherosclerosis, epigenetic, DNA methylation, histone modifications, noncoding RNA modification

## Abstract

Atherosclerosis, characterized by atherosclerotic plaques, is a complex pathological process that involves different cell types and can be seen as a chronic inflammatory disease. In the advanced stage, the ruptured atherosclerotic plaque can induce deadly accidents including ischemic stroke and myocardial infarction. Epigenetics regulation, including DNA methylation, histone modification, and non-coding RNA modification. maintains cellular identity *via* affecting the cellular transcriptome. The epigenetic modification process, mediating by epigenetic enzymes, is dynamic under various stimuli, which can be reversely altered. Recently, numerous studies have evidenced the close relationship between atherosclerosis and epigenetic regulations in atherosclerosis, providing us with a novel perspective in researching mechanisms and finding novel therapeutic targets of this serious disease. Here, we critically review the recent discoveries between epigenetic regulation mechanisms in atherosclerosis.

## 1 Introduction

In recent years, with the fast development of the economy and the change of people’s living habits, cardiovascular diseases (CVDs), including coronary heart diseases and cerebrovascular diseases, have seriously threatened human health in modern society (Bufalino et al., 2020). Even huge progress has been achieved in preventing and treating CVDs, CVDs are still one of the main causes of human mortality and morbidity worldwide. According to one survey conducted by American Heart Association in 2021, the prevalence of CVDs (including chronic heart diseases, Heart Failure, and stroke) in adults ≥20 years is 9.3% overall and increases with age in both males and females (Virani et al., 2021). Among all the causes of CVDs, atherosclerosis accounts for one of the primary underlying causes with the characteristics of narrowing the blood vessels.

Till now, the pathology of atherosclerosis remains unclear, and the risk factors for atherosclerosis include oxidative stress reaction, chronic inflammation, lipids dysmetabolism, genetic factors et al. (Zhong et al., 2019; Leong, 2021). Epigenetics mediates transcriptomic modification without changing DNA sequences and this process is responsive to environmental stimuli (Napoli et al., 2021). Researchers in recent decades have confirmed that aberrant epigenetic modifications participated in the initiation and progression of atherosclerosis (Lee et al., 2020; Karthika et al., 2021; Wong et al., 2021). In this review, we will focus on illuminating the regulation mechanisms between epigenetic modification and atherosclerosis pathology.

## 2 The Pathology of Atherosclerosis Involves Multiple Types of Cells

Atherosclerosis is the result of lipid metabolism disorder in the body, the disbalance of influx and efflux of lipids, especially for low-density lipoprotein (LDL) and oxidized low-density lipoprotein (Ox-LDL), gradually leads to chronic inflammation in blood vessels (Poznyak et al., 2021). The pathological process of atherosclerosis involves multiple types of cells and can be seen as the chronic inflammatory process of the blood vessel wall (Khan et al., 2021). The accumulation of LDL and Ox-LDL leads to endothelial cell (EC) dysfunction, and further, the activated ECs subsequently recruit the monocytes to the damaged site, then monocytes differentiate into macrophages (Xu et al., 2021a; Malekmohammad et al., 2021). The differentiated macrophages and resident macrophages take up lipoprotein forming the initial foam cells (Mushenkova et al., 2021). At the same time, the inflammatory macrophages secret and recruit a series of cytokines, such as interleukin 1 (IL-1) and chemoattractant protein 1(MCP-1), and this mechanism provides a theoretical basis for anti-inflammation therapy in atherosclerosis treatment (Zhang et al., 2021a). In addition, the clearance efficiency of apoptotic cells in atherosclerosis plaque is much lower owing to the impaired efferocytosis, which also contributes to atherosclerotic plaque formation (Wang et al., 2020). In the primary stage of atherosclerosis, atherosclerotic plaques are lying underneath the blood vessel and keep in a stable state with the help of the extracellular matrix. The deposition of plaque gradually narrows the blood vessels, contributing to ischemic diseases. The thrombus also promotes plaque healing process, which leads to progressive intimal thickening and aggravates downstream artery stenosis (Libby, 2021).

## 3 Epigenetic Regulation of Atherosclerosis

The chromatin mainly consists of DNA and histone proteins (H2A, H2B, H3, and H4), which determine gene transcription and transduction. Although DNA and histone proteins are highly packaged, the environment of chromatin is not stereotyped and the structure of chromatin is dynamic when subjects to various stimuli and modifications (Bhutani et al., 2011). Subsequently, the change of chromatin affects information transduction to RNA, which further alters the expression of certain proteins. The epigenetic, in brief, can be described as potentially inheritable chromatin regulation without altering the primary DNA sequence. The occurrence of epigenetics can be ascribed to the interaction between environment and gene (Baccarelli and Bollati, 2009; Burdge and Lillycrop, 2010). Epigenetics are mainly composed of three aspects: DNA/RNA methylation, post-translational modification (PTM) of histone proteins, and non-coding RNA modification (Xu et al., 2019).

The main process of DNA/RNA methylation and histone modifications briefly consist of “readers”, “writers”, and “erasers”. Epigenetic “readers” are specialized domains detecting methylated histone (Zhang et al., 2021b; Zhang et al., 2021c). Epigenetic “writers” include DNA methyltransferases (DNMTs), histone acetyltransferases (HATs), histone methyltransferases (HMT). Epigenetic “erasers” include ten-eleven translocation (TET) methylcytosine dioxygenases, histone deacetylases (HDACs). The coordination of epigenetic readers, writers, and erasers regulates gene expression either by active epigenetic marks or repressive epigenetic marks towards target gene promoters (Xu et al., 2021b). Non-coding RNA refers to RNA that cannot be translated into protein, and in the past, noncoding RNA was considered to be insignificant in gene transcription. However, in the near decades, non-coding RNA (ncRNA) was evidenced to exert different regulating functions in this process (Xu et al., 2019).

Studies suggested that epigenetic regulation exerts a pivotal role in many diseases such as cancers and CVDs (Liu et al., 2021; Yang et al., 2021). Distinct from genetic alteration, epigenetic modification is reversible, and the inhibitor of epigenetic alteration provides a novel insight into searching pharmaceutical therapeutic targets (Pons et al., 2009). Many studies have revealed the pivotal roles that epigenetic modification played in atherosclerosis progression and plaque rupture, and techniques such as chromatin immunoprecipitation sequencing (ChIP-seq) and epigenomic mapping techniques based on genome make it possible to analyze the epigenetic diversity between normal and atherosclerotic tissue (Yue et al., 2014; Lee et al., 2020). In the following part, we will discuss the mechanism of epigenetic modifications in atherosclerosis in detail.

### 3.1 DNA/RNA Methylation in the Pathology of Atherosclerosis

Among epigenetics, DNA methylation is the most researched in the mechanism of atherosclerosis. Methylation and demethylation regulate gene translation and transduction, which means DNA methylation is dynamic and can be modified or reversed, so is the structure of DNA. And generally, the methylation of the promoter is regarded as the marker of the silenced gene. According to different functions in DNA methylation, DNMTs can be classified into three types: DNMT1, DNMT3a, and DNMT3b. For specific, DNMT1 is responsible for maintaining DNMT by adding methyl to hemi-methylated DNA in mitosis when cell replicating. And DNMT3a and DNMT3b add methyl groups to unmethylated DNA directly from *de novo* in mammals (Jeltsch and Jurkowska, 2014). The significant role of DNA methylation played in atherosclerosis is reported by many studies (Zhang et al., 2021b; Jeong et al., 2021). Zaina et al. studied the DNA methylation of atherosclerosis in humans and mapped the DNA methylation profile in atherosclerosis, they reported that DNA methylation regulated different inflammatory genes and pathways (Zaina and Lund, 2014). Valencia et al. interrogated atherosclerotic and normal aortic samples and they found atherosclerosis progression-specific DNA hypermethylation profiles in the vascular tissue (Valencia-Morales et al., 2015).

Atherosclerosis is a chronic inflammatory disease in blood vessels characterized by infiltration of inflammatory cells and cytokines. The irregular shear stress plus lipid dysmetabolism initially activate the ECs, subsequently, the monocyte, macrophage, and vascular smooth muscle cell (VSMC) are activated and recruit inflammatory cytokines such as MCP-1, matrix metalloproteinase (MMP), and tumor necrosis factor *α* (TNF-α) (Sugiyama et al., 2001; Ding et al., 2015). In recent years, studies have revealed the key roles DNA methylation played in the inflammation of atherosclerosis (Shuto et al., 2006; Ianni et al., 2013). Stenvinkel et al. analyzed DNA methylation of peripheral blood leukocytes in chronic kidney disease and they found that global DNA hypermethylation was correlated with inflammation reaction, which contributed to the high mortality in chronic kidney disease (Stenvinkel et al., 2007). The dysmetabolism of lipid is one the of main causes of atherosclerosis, and among lipoproteins, dysmetabolism of Ox-LDL is key in atherosclerosis. Ding et al. reported the role of Ox-LDL lipoprotein played in inflammation in vascular ECs. In their study, they also found that DNA methylation in inflammation is one of the potential risk factors in CVDs (Ding et al., 2015). Lectin-like oxidized low-density lipoprotein receptor-1 (LOX-1) recognizes and regulates the metabolism of ox-LDLs in ECs. Tian et al. reported that DNA hypomethylation is associated with ECs damage, and they confirmed this effect was mediated through toll-like receptor 4 (TLR4)/nuclear factor-kappa B (NF-κB) (Tian et al., 2018). Nitric oxide synthase (NOS) is synthesized in ECs, NOS exert a significant role in inflammation of atherosclerosis (Forstermann and Sessa, 2012). Heiss et al. suggested that posttranslational modifications of NOS could be regulated through DNA methylation (Heiss and Dirsch, 2014). Kruppel-like factor 2 (KLF2) is one of the critical anti-inflammatory mediators in atherosclerosis in ECs, which is also the transcriptional factor of NOS (Atkins et al., 2008). Inflammatory molecular lipopolysaccharides (LPS) regulate the expression of KLF2 through DNA hypermethylation, and this discovery provided a novel perspective to atherosclerosis (Yan et al., 2017).

Irregular blood shear stress is one of the potential risk factors to endothelium injury, which contributes to the pathology of blood vessel atherosclerosis (Björck et al., 2018). Atherosclerosis plaques are prone to develop in arteries where blood flow is disturbed. Dunn et al. found that gene expression of ECs could be altered in such conditions, and they evidenced that the expression level of DNMT was increased in cultured ECs when suffering oscillatory shear stress (Dunn et al., 2014). Jiang et al. found that disturbed blood flow increased DNA methylation within the KLF4 promoter, which further lead to suppression of KLF4 expression. And this effect could be mitigated by utilizing DNMT inhibitors (Jiang et al., 2014). Zhou et al. also found that in rat carotid arteries, oscillatory shear flow induces DNA hypermethylation, which exerted a role in the injury of ECs, and this effect was mediated by DNMT manner (Zhou et al., 2014).

The effect of DNA methylation is mediated through DNMT, and different DNMTs may play different roles in atherosclerosis (Yu et al., 2012; Yang et al., 2014). Deng et al. found that the expression level of DNMT1 was correlated with the expression level of monocytes and DNA hypomethylation was independently linked with the risk of CADs in the Chinese population (Deng et al., 2018). The role of DNMT1 played in atherosclerotic plaque was also evidenced in other studies (Greissel et al., 2015). Yu et al. reported that DNMT 3a limited the expression of interleukin-13 in T helper two cells, which further inhibited inflammation reaction (Yu et al., 2012). Yang et al. suggested that DNMT 3b regulated macrophage polarization and inflammation through DNA methylation at promoter location (Yang et al., 2014).

Besides DNMT dependent manner, DNA demethylation also can be mediated through TET, which includes TET1, TET2, and TET3. Greissel et al. found that the expression of DNA-demethylase TET1 was increased in atherosclerotic plaques, which was consistent with DNA hypomethylation (Greissel et al., 2015). Liu et al. suggested the significant role of TET2 played in the phenotype transformation of vascular smooth muscle cells, dysfunction of endothelial cells, and macrophage inflammation, which contributed to the pathology of atherosclerosis (Liu et al., 2018). Jiang et al. suggested that DNA demethylation mediated by TET3 played a key role in DNA injury repairmen and stability of gene expression (Jiang et al., 2017).

Besides DNA methylation, RNA methylation is a newly emerging field of epigenetics in atherosclerosis. Fu et al. reported the relationships between N6-methyladenosine (m6A) modification in messenger RNA (mRNA) and atherosclerosis risk factors (Fu et al., 2021). Chien et al. identified m6A methyltransferase-like 3 (METTL3) as a responsive hub when subject to hemodynamic forces, which was atherogenic stimuli in endothelial cells (Chien et al., 2021). Similarly, METTL3-dependent m6A methylation in regulating inflammatory processes was also reported in Zhang et al. study, which contributed to a more comprehensive understanding of atherosclerosis pathogenesis (Zhang et al., 2021d).

### 3.2 Histone Modification in the Pathology of Atherosclerosis

Compared to DNA methylation, PTM is more complex, which occurs at the N-terminal of histones and plays a crucial role in regulating gene expression (Bentzon et al., 2014). The function of PTM is dependent on the modification of different residues and varies among different tissues. The PTM mainly includes acetylation, methylation, phosphorylation and ubiquitination, SUMOylation, GlcNAcylation. Among PTM, histone acetylation and methylation are the most researched, which are closely associated with the inflammatory process of cardiovascular diseases (Zhang et al., 2014). Histone acetylation is mediated by HATs and histone deacetylases (HDACs) while histone methylation is mediated by histone methyltransferases (HMTs) (Pons et al., 2009; Jiang et al., 2018).

#### 3.2.1 Histone Acetylation and Pathology of Atherosclerosis

Histone acetylation, mediating *via* HATs, is defined as adding positive acetyl groups to amino acid residues of histones, which reduces the combination between histone protein and DNA and further activates gene expression. Histone deacetylases, mediating via HDACs, refers to the removal the acetylated residues at lysine and further inhibits gene expression (Chen et al., 2020). Recently, various studies have confirmed the key role HATs and HDACs played in the pathology of atherosclerosis. Hoeksema et al. suggested that HDAC3 exerted a significant role in the phenotype of macrophages in atherosclerotic lesions, and collagen deposition was observed in HDAC3 deletion mice, promoting the stabilization of atherosclerotic plaques (Hoeksema et al., 2014). Mullican et al. found that in HDAC3 deletion mice, the expression of the IL-4 gene was elevated, and the alternative anti-inflammatory cytokine secretion was evidenced in macrophages (Mullican et al., 2011). Lee et al. suggested that HDAC groups exerted key roles in oxidative and inflammatory reactions, in their study, they found that under disturbed blood flow, the expression of HDAC2, HDAC3, HDAC5 was elevated in the rat aortic arch, which resulted in a pro-atherogenic phenotype of endothelial cell (Lee et al., 2012). Li et al. found that HDAC1 was reduced in atherosclerotic lesions, mediating ECs apoptosis by miR-34a (Li et al., 2018). Sun et al. reported that HDAC1 was pivotal in the migration and phenotypic transformation of VSMCs (Sun et al., 2020). Thus, the role of HDACs played in atherosclerosis is complex, the multiple functions may vary depending on the different cell types.

While controversial roles exist in HDACs, the role of HAT is considered to be proatherogenic. Kawahara et al. observed the hyper-nuclear acetylation in VSMCs of coronary atherosclerotic lesions (Kawahara et al., 1999). Greissel et al. found in human carotid plaques, histone acetylation was localized in ECs, VSMCs, and macrophages (Greissel et al., 2016). Inhibiting HAT expression was accompanied by increased cholesterol efflux in macrophages, this anti-inflammatory effect was mediated by decreased NF-kB activation (Lin et al., 2015).

#### 3.2.2 Histone Methylation and Pathology of Atherosclerosis

Histone methylation refers to transferring a methyl group from S-adenosyl-l-methionine to lysine or arginine residues in histone via HMTs, in this process, the charge of histone protein keeps unchanged. Compared to histone acetylation and deacetylation, histone methylation is a more complex process, the modification of different residues in histone may lead to activating or repressive chromatin configuration. Also, the amount of methyl group added to histone residues may vary from mono-methylation, di-methylation, and tri-methylation (Bannister and Kouzarides, 2011). Among them, histone three lysine 27 trimethylation (H3K27me3), histone three lysine nine trimethylation (H3K9me3), and histone three lysine four trimethylation (H3K4me3) are typical epigenetic marks of histone methylation.

The association between histone methylation and atherosclerosis was revealed in many studies. Alkemade et al. confirmed that in Apoe–mice, the H3K27me3 level was reduced in VSMCs (Alkemade et al., 2010). Wierda et al. studied the relationship between H3K27me3 modifications and atherosclerotic plaque in human renal aortic tissue, the immunohistochemistry result found that the expression level of H3K27me3 was reduced in atherosclerotic blood vessels, which was related to the VSMCs migration and proliferation (Wierda et al., 2015). Bekkering et al. isolated human monocytes and exposed them to oxLDL, after 24 h, the elevated expression of H3K4me3 of inflammatory genes include MCP-1, TNF- α, IL-6, IL-18, MMP was observed, which further exerted a proatherogenic role *in vitro* (Bekkering et al., 2014). Jumonji C domain protein (JMJD) expressed in macrophages is a specific H3K27me3 demethylase and polycomb group (PcG) protein that can mediate gene silencing. De et al. found that in macrophages JMJD3 could regulate H3K27me3 levels by binding PcG in response to inflammation and transcriptional activity. When exposed to lipopolysaccharide (LPS), the levels of JMJD3 were increased in macrophages (De Santa et al., 2007).

### 3.3 Non-coding RNA Modification in the Pathology of Atherosclerosis

Besides DNA methylation and histone modifications, there are more and more studies confirming the existence of ncRNA epigenetic modifications in the pathology of atherosclerosis (Wang et al., 2019; Chang and Wang, 2021). According to the amounts of base pairs, ncRNAs can be classified into small ncRNAs such as microRNAs (miRNA), long ncRNAs such as long intergenic ncRNA (lncRNA), circular RNA (circRNA) (Boon et al., 2016).

#### 3.3.1 miRNA in the Pathology of Atherosclerosis

MiRNA, consisting of 20–40 nucleotides, regulates post-transcriptional gene expression. In many diseases, this process is accompanied by DNA methylation or histone modifications (Tao et al., 2021). miRNA broadly participates in inflammation, oxidative reaction, cholesterol balance, and all of these processes are atherosclerosis risk factors. The specific mechanism of miRNA contributing to atherosclerosis varies depending on the cell types (Hosen et al., 2020). Leeper et al. summarized the key role miRNA played in VSMCs in atherosclerosis, and they suggested that miRNA could potentially be a biomarker when diagnosing and treating this disease (Leeper and Maegdefessel, 2018). Other studies also revealed the importance miRNAs played in endothelial dysfunction and macrophage failure in atherosclerosis (Schober and Weber, 2016; Poller et al., 2018).

#### 3.3.2 lncRNA in the Pathology of Atherosclerosis

LncRNA consists of more than 200 nucleotides, in a long time, lncRNA was considered to be insignificant in gene expression. Similar to miRNA, lncRNA can exert epigenetic roles in different cell types in atherosclerosis. Guo et al. reported that LncRNA PVT1 knockdown alleviated ECs injury and atherosclerosis in mice (Guo et al., 2021). Weng et al. suggested that LINC01123 promoted cell proliferation and migration through regulating miR-1277-5p in VSMCs, which may be involved in fibrous plaque formation (Weng et al., 2021). Huang et al. reported in macrophage, lncRNA H19 overexpression significantly increased the atherosclerotic plaque in mice via the miR-146a-5p pathway (Huang et al., 2021a).

#### 3.3.3 circRNA in the Pathology of Atherosclerosis

CircRNAs are closed circular molecules, which are generated from splicing of specific regions of pre-mRNAs (Bayoumi et al., 2018). More recently, growing studies have shown regulating effects of circRNAs in atherosclerosis (Cao et al., 2020). Huang et al. found circUSP36 promoted EC dysfunction in atherosclerosis via miR-637 (Huang et al., 2021b). Li et al. reported in the serum of atherosclerosis patients, circ_0002984 was elevated and further study confirmed its role in the proliferation and migration of VSMCs (Li et al., 2021). And in He et al.’s study, circSCAP aggravated macrophage injury in atherosclerosis (He et al., 2021). In brief, the newly emerging circRNAs have broadened our understanding of atherosclerosis pathology.

## 4 Conclusion

Till now, a growing number of studies have revealed the relationship between epigenetic regulation and atherosclerosis. However, owing to the complexity of the pathology of atherosclerosis, the current discoveries are still in a preliminary stage. Epigenetics transfer and translate environmental stimuli of atherosclerosis risk factors into reversible gene abnormal expression. The improvement of epigenetic analysis technology, as well as more efforts by researchers, will significantly increase our understanding of the potential epigenetic marks either as diagnostic or as a therapeutic agent in atherosclerosis.

Our review briefly summarizes the regulatory mechanism of epigenetic modifications in different stages of atherosclerosis progression and different cell types. Bedside the questions, improvements have been acquired in the pharmacology of targeted drugs to inhibit atherosclerosis progression. Although most of the therapeutic agents currently are still in the laboratory stage, it can be envisaged that as the understanding of epigenetic modification is deepening, novel therapeutic interventions of atherosclerosis are in the near corner.
